# Sponge Morphology of Osteosarcoma Finds Origin in Synergy Between Bone Synthesis and Tumor Growth

**DOI:** 10.3390/nano15050374

**Published:** 2025-02-28

**Authors:** Arnaud Bardouil, Thomas Bizien, Jérome Amiaud, Alain Fautrel, Séverine Battaglia, Iman Almarouk, Tanguy Rouxel, Pascal Panizza, Javier Perez, Arndt Last, Chakib Djediat, Elora Bessot, Nadine Nassif, Françoise Rédini, Franck Artzner

**Affiliations:** 1 CNRS, Institut de Physique de Rennes (IPR), UMR 6251, Université de Rennes, 35000 Rennes, France; 2 Synchrotron SOLEIL, L’Orme des Merisiers, 91190 Saint-Aubin, France; 3 INSERM, UMR 1307, Team CHILD, Nantes University, 44035 Nantes, Franceseverine.battaglia@univ-nantes.fr (S.B.); francoise.redini@univ-nantes.fr (F.R.); 4 INSERM, UMR 991 Liver Metabolism and Cancer, Rennes University, 35000 Rennes, France; 5 Karlsruhe Institute of Technology, Institute of Microstructure Technology, 76344 Eggenstein-Leopoldshafen, Germany; 6 CNRS, Muséum National d’Histoire Naturelle, UMR 7245, Bâtiment 39, CP 39, 57 rue Cuvier, 75231 Paris, France; 7 CNRS, Sorbonne Université, Collège de France, Laboratoire Chimie de la Matière Condensée de Paris (LCMCP), 75005 Paris, Francenadine.nassif@sorbonne-universite.fr (N.N.)

**Keywords:** X-ray diffraction, bone, hydroxyapatite, simulation, hierarchical structure

## Abstract

Osteosarcoma is medically defined as a bone-forming tumor with associated bone-degrading activity. There is a lack of knowledge about the network that generates the overproduction of bone. We studied the early stage of osteosarcoma development with mice enduring a periosteum injection of osteosarcoma cells at the proximal third of the tibia. On day 7 (D7), tumor cells activate the over-synthesis of bone-like material inside the medulla. This overproduction of bone is quickly (D13) followed by degradation. Samples were characterized by microfocus small-angle X-ray scattering (SAXS), wide-angle X-ray scattering (WAXS), optical and electron microscopies, and micro-indentation. This intramedullary apatite–collagen composite synthesis highlights an unknown network of bone synthesis stimulation by extramedullary osteosarcoma cells. This synthesis activation mechanism, coupled with the well-known bone induced osteosarcoma growth activation, produces a rare synergy that may enlighten the final osteosarcoma morphology. With this aim, a 3D cellular automaton was developed that only included two rules. Simulations can accurately reproduce the bi-continuous sponge macroscopic structure that was analyzed from mice tumor micro-tomography. This unknown tumor activation pathway of bone synthesis, combined with the known bone activation of tumor growth, generates a positive feedback synergy explaining the unusual sponge-like morphology of this bone cancer. From a biomaterials point of view, how nature controls self-assembly processes remains an open question. Here, we show how the synergy between two biological growth processes is responsible for the complex morphology of a bone tumor. This highlights how hierarchical morphologies, accurately defined from the nanometer to the centimeter scale, can be controlled by positive feedback between the self-assembly of a scaffold and the deposition of solid material.

## 1. Introduction

All natural materials exhibit complex and hierarchical organization that provides an unlimited source of inspiration in material chemistry [1]. A large variety of inorganic compounds are shaped at ambient temperatures with characteristic dimensions and morphologies spanning from the nanometer to the centimeter. These materials, such as bone, nacre, and sponges, are sophisticated composite phases of organic/biological templates and hydroxyapatite, calcium carbonate, or silica. Although the main polymeric components (proteins, …) are becoming better and better identified, the fundamental mechanisms compatible with well-defined organizations, from the nanometer and to centimeter scale, remain a mystery. This is mainly due to the huge experimental difficulty in simultaneously characterizing the structures of both inorganic materials and organic templates. Most suitable techniques require destructive steps such as fixation, staining, or cutting, which makes it impossible to study the detailed building mechanisms alive.

From a theoretical point of view, organizational building results mainly from two elementary processes, which are (i) organic template self-assembly and (ii) mineralization. Pivotal question exist regarding the coupling between these processes, such as its existence and its nature (chemical or biological, …). A lack of coupling between these elementary steps would typically result in chaotic structures. Among the large number of biological and bioinspired materials investigated [1], an ultimately simplified system demonstrated that synergy between a self-assembled dynamical template and a mineralization process can generate double-wall silica nanotubes with a silica thickness accuracy smaller than the nanometer scale and nanotube length longer than the centimeter scale [2]. To make such multiscale spontaneous organization, the product of one self-assembly process must locally activate the other self-assembly “reactions”: (i) the cationic surface of the scaffold catalyzes the silica polymerization; (ii) the negative silica charges neutralize the reaction site and favor the monomer self-assembly by reducing Coulomb repulsions with the tip of the in-construction nanotube. The synergy between both reactions perfectly sets up the alternation of both template and mineralization delivery, similarly to the architect scheduling the sequential supply of concrete and steel during skyscraper building. This synergy mechanism was assumed to be a potential coupling mechanism that explains the morphology of biological silica spicules [3]. This mutual coupling between self-assembly and mineralization processes was then proposed for various inorganic materials, such as metallophosphates [4] or silica [5] with lipids, as well as calcite in sea urchin spicules [6], foraminifera [7], and interactions with silk fibroin [8]. It is also compatible with the formation of collagen/apatite composite in bones [9]. Three-dimensional crystals of Quantum Dots were similarly manufactured by synergy between lipid vesicle disruption and the biological polymerization of actin proteins [10]. Such synergy between dynamic self-assemblies is expected to occur at any scale, and we anticipate here that sponge-like bone structures of a rare bone cancer, osteosarcoma, may find their origins in such a mechanism.

Bone is a dynamic tissue in a state of constant remodeling, which involves a multi-step process including the commitment of various cell types. Osteocytes [11,12] are sensitive to local pressure and may activate the bone resorption process via the osteoclasts, which locally dissolve bone matter, releasing elements from the bone matrix and smoothing out the surface for bone construction to occur. Once the bone is satisfactorily resorbed, osteoblasts produce the new bone matrix to renew the local bone structure and adapt it to the different kinds of stress the bone is locally subjected to [13,14]. In this process, some osteoblasts are captured in the bone matrix, being specialized into a new set of osteocytes. The entire system of bone exists in some form of dynamical equilibrium, remodeling bone to best fit the stresses applied to it, or as a means of the storage and release of some minerals [12]. Bone production is also a phenomenon that appears in the natural growth of non-mature individuals [15].

Osteosarcoma represents the most frequent malignant primary bone tumor that predominantly arises in adolescent and young adults (median 18 years) [16,17]. It appears as a bone-forming tumor in X-ray imaging, but the presence of bone osteolysis areas are also observed [18]. The abundant osteoid matrix formation inside the tumor tissue specific to osteosarcoma is then confirmed by pathologists. The concept of a vicious cycle between tumor cell proliferation and bone degradation has emerged from previous preclinical analyses [16]; osteosarcoma cells produce cytokines, such as interleukin-1 or -6, that activate osteoblasts to produce the cytokine Receptor Activator of NF-kB Ligand (RANKL), which regulates the osteoclast activity, leading to bone degradation. When they degrade bone, osteoclasts allow the release of growth factors, which in turn will activate tumor cell proliferation. Besides this well-described vicious cycle, nothing is known about the bone-forming activity of osteosarcoma cells, neither in terms of the quality of the bone formed, nor on its production kinetics. In particular, nothing is known about collagen structuring or the mineralization activation of this tumoral osteoid matrix.

The sponge-like morphology observed in osteosarcoma [18,19] remains an open question. There is a lack of knowledge about collagen structuring and mineralization activation in osteosarcoma. The newly formed bone observed in this tumor is usually described as “immature bone”, or “tumor osteoid matrix” (refs). Such complex patterns always find their origin in coupled reaction networks [20,21]. The historical explanation was the prey–predator model from Turing [22,23]. The spatial instabilities can come from a network of reactions (chemical, cell growth or death, …). In this spirit, we may expect that the entangled topologies of the bone and tumor cell growths should find their origin in a network of loops and growths with positive feedback, i.e., synergy. At the current stage of osteosarcoma growth knowledge, the bone is known to enhance the sarcoma cell division, but no mechanism has been elucidated concerning the activation of bone growth by tumor cells [24].

In this paper, we investigate this complex network using a preclinical murine model of osteosarcoma (i) by studying bone growth induced by tumor cells at early stages, and (ii) by comparing osteosarcoma sponge-like morphologies observed by microtomography with simulations and a minimal network of loops and positive feedback. The first part of the paper is dedicated to the demonstration of long-range bone formation activation by osteosarcoma cells via steady-state experiments. For this purpose, osteosarcoma cells were inoculated extracortically in close contact with the mouse tibia [25], leading to a fast response via de novo intracortical bone overproduction. The bone ultrastructure was analyzed in depth using X-ray scattering [26,27,28], electron microscopy, and mechanical properties. The second part of the paper is focused on modeling the sponge-like morphology of osteosarcoma by a minimal network of cross-reaction. This is achieved by considering only two reactions: tumor growth and bone growth. The activation network is reduced to a positive feedback, with tumor invasion favored by bone proximity and bone growth favored by tumor proximity. Three-dimensional simulations are in good agreement with the sponge-like morphology observed by micro computed tomography (micro-CT) in the osteosarcoma final stage.

## 2. Materials and Methods

### 2.1. Syngenic Model of Osteoblastic Osteosarcoma in Mouse (MOS-J) and Cryosection

Mouse Osteogenic Sarcoma-Jackson (MOS-J) mouse osteosarcoma cells (4.106) were inoculated in C57.Bl/6J mice (male, aged 5 weeks) by intramuscular injection in close contact with the tibia. Mice were housed at the experimental therapeutic unit of the Nantes medical school (France). All animal experiments were realized in agreement with the protocols of the regional committee of animal experiments, CEEA, n°6 Pays de la Loire. Tumors appeared at the injection site approximately 8 days later, leading to both osteoblastic and osteolytic lesions that reproduced the clinical form of human osteoblastic osteosarcoma.

In this experiment, the mice infection process starts with the opening of the left hindleg at about the third proximal of the tibia. The exposed bone, the periosteum, is then scratched with a metallic spatula to activate bone remodeling for the osteosarcoma to subvert better. 4.5 M MOS-J osteosarcoma cells are then deposited through 50 μL of solution upon the scratched surface before closing the cut. For the control sample, the same process is repeated upon the right hindleg, except for sarcoma cell deposition. This induces intense bone modeling between the proximal metaphysis and the diaphysis. When the periosteum is destroyed because of tumor aggressiveness, osteogenesis occurs perpendicular to the long axis of the bone, with the periosteal appositions having the appearance of spicules [29,30]. This process extends rapidly in the soft parts. MOS-J cells also have migration potential, leading to lung metastases development.

A pool of seven different subjects was prepared for this study. Three pairs were sacrificed at 2, 7, and 13 days of development for the early-stage part, with one more for late-stage osteosarcoma. Half the early-stage samples were decalcified for optical microscopy purposes. The control samples pool is made of healthy contralateral tibias from the animals. The number of sacrificed mice was limited due to ethical principles.

In the case of the late-stage sample, tumor volume is measured manually with a caliper all through the experiment to follow the tumor progression in bone. Animals were sacrificed when the volume measurement reached 2000 μm^3^ at 28 days after injection. Tibias injected with MOS-J cells and control contralateral tibias were collected and fixed in formalin before analysis.

**Figure 1:**
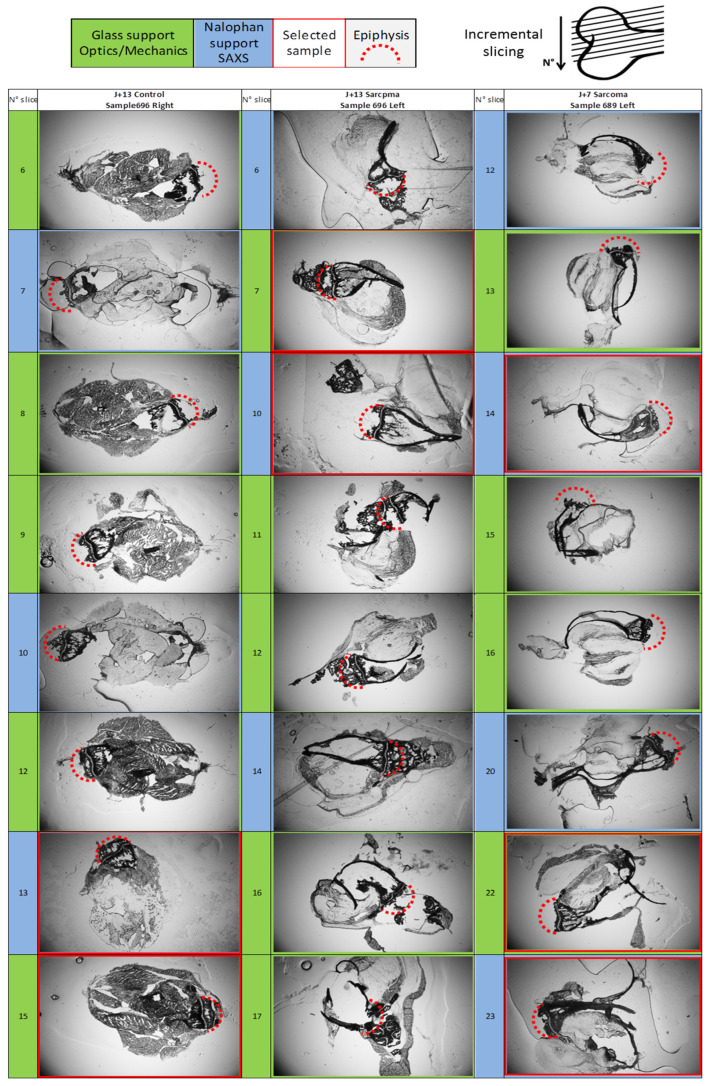
Incremental slices pictures 9.5 mm × 14.3 mm pictures of control, D13, and D7 sample slices. The incremental slices are numbered on the left, with support material and selected samples color-coded. This table allows the comparison of trabecular material in the medium-sized cavity between samples. The location of the epiphysis is suggested with a red-dot half-circle, with the diaphysis opposite from it.

The samples are taken from storage at −80 °C and then glued to a support. Samples are then cut into successive thin layers of approximatively 100 μm thickness using a LEICA CM3050S (Wetzlar, Germany). Layers are deposited alternatively on glass and nalophan supports for mechanical and X-ray diffraction, respectively. Samples are then dried 24 h in an air-flow dryer at 40 °C (Figure 1).

### 2.2. Optical Microscopy of 100 μm Thick Slices

**Figure 2:**
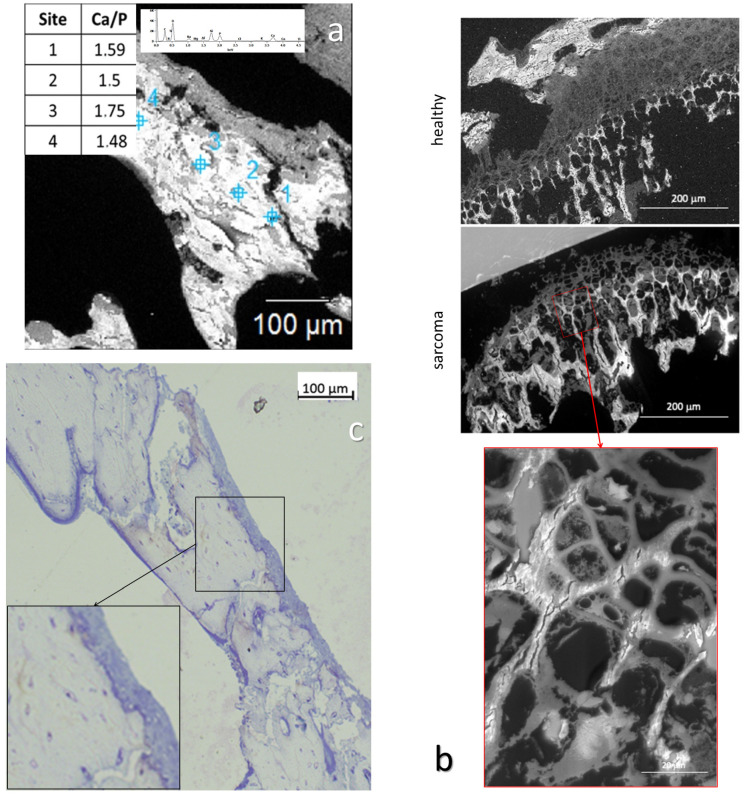
Thin-slice BSE and optical microscopy. (**a**) Back-Scattering Electron microscopy from a D7.2 medullar cavity trabeculae, with different sites molar ratio of calcium and phosphorus, where the inset is a representative EDS spectrum. (**b**) BSE microscopy from healthy and sarcoma-inoculated tissue (D7.2 thin slice). Intensity is relative to the amount of mineralization. Zooming in showcases the creeping mineralization occurring in the inoculated sample’s growth cartilage. (**c**) Optical microscopy image of a thin slice of trabecular bone from D7.2. Colored dots in the bone matrix are osteocytes, while the layer on the periphery of the bone is made of bordering cells and quiescent osteoblasts.

Optical microscopy observations were performed using the IX70 inverted microscope (Olympus, Tokyo, Japan). For comparison, pictures of the samples were taken on a wide-angle camera with a resolution of 5760 × 3840 pixels on a 36 mm × 24 mm receptor. The setup was made from a light source, an (x;y) axis moving plate, and the Canon camera EOS 5D Mark III, and gave us a resolution on picture of 1.2 μm per pixel (Figure 2).

### 2.3. Microfocus SWING Beamline

Most structural data of bone and bone-like material were obtained by performing the SAXS experiment on samples at the SWING beamline of the SOLEIL synchrotron.

Samples were mapped using a SAXS Microfocus setup at 12 keV on the SWING beamline of SOLEIL synchrotron. The microfocus beam was obtained using only horizontal Compound Refractive Lenses (H-CRLs) made of SU8 designed especially for the beamline at Karlsruhe Institute of Technology (KIT). Indeed, a 20 μm size beam (on the sample) can be obtained vertically with the Vertical Focusing Mirror (VFM), and thus only H-CRLs are needed [31,32]. To obtain the micro focused beam, the horizontal focusing mirror (HFM) was removed and H-CRLs were inserted into the beam. H-CRLs for 12 keV are made of 7 elements and possess a physical aperture of 250 μm. Thus, the horizontal guarding slits are closed at a gap of 200 μm. VFM focalization is set so the beam size is 20 μm vertically. All those changes in the beam lead to a 10-fold beam intensity decrease, but thanks to focalization, flux density is increased 3 times. After blade scans of the focused beam, the beam size on the sample is found to be 17 × 17 μm^2^.

Sample scanning was achieved thanks to a raster scan developed at SOLEIL, called “flyscan” [33]. This type of scanning allows one motor to move continuously while another works as a step motor. Motors positions and all other needed data are recorded for each SAXS image recorded. Thus, we record all motors position for each image along a continuous line of scanning. For each sample, maps were obtained using SAXS diffraction patterns linked to each other by their motor positions.

Data were collected using an AVIEX CCD detector, 1024 × 1024 pixels of 166.8 × 166.8 μm^2^ at a sample to detector distance of 674 mm. The image step was 30 microns.

Hydroxyapatite (HA) nanocrystals and type I collagen, as the main bone components, are the main sources of diffracted signal in SAXS.

### 2.4. SAXS Parameters Mapping

We used a panel of different data reduction techniques on the SAXS spectra, whether in 2D or 1D format. A 1D format is the result of the radial integration of 2D spectra with the beam’s incident point as the origin.

Scattered intensity is evaluated by summing the individual values of each pixel of 2D spectra, which are proportional to the number of photons received by the corresponding pixel from the detector upon the acquisition time. The summed pixels are those at a distance from the incident beam origin, corresponding to a q range from 0.01 to 0.045 Å^−1^ (~140 to 630 Å) for the length of calculations, and a detector saturation too close to the origin. The range covered is almost exclusively an apatite signal.

From 2D spectra, it is possible to calculate the values of orientation of signal θ and a value of anisotropy A going from 0 (isotropic) to 1 (perfectly aligned) through the separation of signal in eight regular quadrants of 45°. This allows the following operations:

(1) $$I_{n}=\iint_{45\left(n-\frac{1}{2}\right)}^{45\left(n+\frac{1}{2}\right)}I\left(r;\theta \right)d\theta dr$$

(2) $$\theta ={tan}^{-1}\left(\frac{\left(I_{2}+I_{6}\right)-\left(I_{4}+I_{8}\right)}{\left(I_{1}+I_{5}\right)-\left(I_{3}+I_{7}\right)}\right)$$

(3) $$A=\sqrt{{\left[\left(I_{2}+I_{6}\right)-\left(I_{4}+I_{8}\right)\right]}^{2}+{\left[\left(I_{1}+I_{5}\right)-\left(I_{3}+I_{7}\right)\right]}^{2}}$$

HA crystals are aligned with the collagen structure and possess a length far superior to their width, which, in the reciprocal space of SAXS, results in a 90° difference between the orientation of HA crystals and their signal. This was corrected in the mapping.

The parameters of shape and interface of the HA crystals are estimated from the 1D spectra in a log/log scale in which they appear as linear slopes [34] in a −α.q + β fashion, with I(q) ∝ q^−α^. This measure was obtained as follows:

(4) $$\alpha =\frac{\sum_{q_{1}}^{q_{\frac{n}{2}}}logy-\sum_{q_{\frac{n}{2}+1}}^{q_{n}}logy}{\sum_{q_{1}}^{q_{\frac{n}{2}}}logx-\sum_{q_{\frac{n}{2}+1}}^{q_{n}}logx}\;\mathrm{with}\;q\in \left[q_{0};q_{n}\right]$$

HA crystals’ shape is estimated from the log/log slope value on a q range from 0.042 to 0.073 Å^−1^ (~85 to 150 Å), from which we mapped the parameter. The crystal interface slope follows the same process on a range from 0.15 to 0.25 Å^−1^ (~25 to 42 Å).

The perimeters of scattering objects can be determined through the use of Kratky plots, q^2^I(q), where the q coordinate of the maximum value corresponds to the perimeter of the object. Kratky plots thus allow us to follow the perimeter of HA crystals in the slices.

The collagen SAXS signal can be divided between the lateral packing signal of the quasi-hexagonal structure and the longitudinal packing signal (i.e., the 65 nm bands). 

The known peak associated with collagen around 0.5 Å^−1^ is found alongside another one at around 0.7 Å^−1^. These signals correspond to, respectively, ~12 Å and ~8.5 Å. Considering the combined effect of not knowing any other possible structure of bone composition at those interdistances and the correlated presence of and orientation with the lateral packing collagen peak, we consider collagen packing to be responsible for that signal at 0.7 Å^−1^.

We decided to follow the evolution in samples of the peaks’ position and amplitude. The position of the peak is determined through the second derivative of the smoothed signal, with the q coordinate with the lowest value corresponding to the peak position. The amplitude of peaks is measured at the previously determined coordinate after the subtraction of a polynomial fit of the background signal.

### 2.5. Martens Hardness Indentation

Martens hardness measurements, conducted through micro-indentation, were performed on a Fischerscope H100C Sindelfingen, Germany with a Vicker Diamond tip with a 136° face angle. The process is controlled through the software WIN-HCU 3.0, which also automatically measures the Martens hardness parameter of materials though the force applied and the depth of indentation.

### 2.6. Wide-Angle X-Ray Scattering

Wide-angle X-ray scattering results were collected with a Pilatus 300 K (Dectris, Baden-Daettwil, Switzerland), mounted on a microsource X-ray generator GeniX 3D (Xenocs, Grenoble, France) operating at 30 W. The monochromatic CuKα radiation was λ = 1.541 Å. The results were recorded in the reciprocal space in a range from 0.1 to 2.5 Å^−1^, with a distance between sample and detector of 129.3 mm.

### 2.7. Thin Section for Microscopies

Thin sections of bone samples were prepared following the classical procedure for biological tissues. Briefly, glutaraldehyde fixation and post-fixation with 1% osmium tetroxide were performed for 1 h at 4 °C. The samples were then washed in a cacodylate/saccharose buffer solution (0.05 M/0.6 M, pH 7.4), dehydrated through successive ethanol baths, and embedded in Epon. Thin sections (~500 nm) were obtained with an ULTRACUT 7 (Leica, Wetzlar, Germany), deposited on a glass slide, and stained with Toluidine blue (Toluidine blue 1%, sodium borate 1%) for observations by optical microscopy or directly observed by SEM (BSE mode).

Optical microscopy (Figure 2c) was performed using a transmission Zeiss Axio Scope, equipped with an Axiocam 305 camera. The images were processed using the ZEN software 3.11 (Zeiss, Oberkochen, Germany).

For SEM, the thin sections of bone samples (0.5 μm thick) were placed on a carbon tape (12 mm) on top of an SEM sample holder. The observations were performed on a Hitachi SU3500 machine with an accelerating voltage of 10 kV (Figure 2a,b).

### 2.8. Advanced Osteosarcoma MicroCT

Micro computed tomography (Micro-CT) is a 3D imaging technique that uses X-rays to observe, slice by slice, the inside of an object [23,25].

Analyses of the bone microarchitecture were performed using a high-resolution X-ray micro–computed tomography Skyscan 1076 in vivo (Skyscan, Kontich, Belgium). All tibias were scanned using the same parameters (pixel size 9 μm, 50 kV, 0.5 mm Al filter, 30 min of scanning). Reconstruction was performed using the NRecon 2.0 software, reconstructing the structure of an osteosarcoma on a mouse hind leg as stacks of 2D images.

### 2.9. Simulation

#### 2.9.1. Topology Modeling

A reproduction of the osteosarcoma topology was attempted through simulation, in the hope that a faithful reproduction of the structure by the application of development rules would offer leads into the osteosarcoma growth process and mechanisms.

We reduce the system to three species: bone, sarcoma, and healthy tissue. We base the propagation model of bone/sarcoma cells on different hypotheses:

- The structures formed are not static and subject to remodeling. Sarcoma s and bone cells b (p type cells) propagate at speed $\vec{v_{p}}$.
- Bone and sarcoma are the two cell types capable of propagation at the interface in this simulation, while healthy tissue is incapable of it, since it would correspond to tumor regression, which is not considered here.
- Propagation of bone (*b*) and sarcoma (*s*) are each exclusively dependent on the presence of the other. As such, $\vec{v_{b}}$ depends on the presence of *s* and $\vec{v_{s}}$ depends on the presence of *b* as both elements progress together.
- Propagation happens through contact, but under a sum influence exerted at a distance, as the model we are trying to ascertain is one of the propagation being enabled by the diffusion of a mediator. Sum influence takes the form of $\vec{v_{s}}\left(i;j\right)=\iint_{S}\;f\left(d\right)\gamma_{b}\left(i^{\prime };j^{\prime }\right)dS$ with (*i*;*j*) coordinates of cell propagation. *d* is the distance to (*i*;*j*), *γ_p_*(*i*′;*j*′) a binary function of presence of species *p* at coordinates (*i*′;*j*′). *f*(*d*) is a law of distance influence, such as the distance of maximum influence, and the ratio between peak influence and long range can be adjusted.
  (5) $$f\left(d\right)=\frac{1}{{\left(d^{2}+d_{0}^{2}\right)}^{\eta }}$$
  where *d*_0_ is the distance of maximum influence and *η* is long-range influence.
- The resulting domains must be cohesive, and interface roughness in the system is controlled by limiting growth that creates too much interface and clearing out all isolated cells. The limiting function appears in, for example, $\vec{v_{b}}\left(i;j\right)=\vartheta_{b}\left(i;j\right)\iint_{S}\;f\left(d\right)\gamma_{s}\left(i^{\prime };j^{\prime }\right)dS$ with the binary function $\vartheta_{s}\left(i;j\right).$ If there is a function $\rho_{s}\left(i-1;j-1\right)=\sum_{i-1}^{3}\gamma_{s}\left(i\prime ;j^{\prime }\right)+\sum_{j-1}^{3}\gamma_{s}\left(i^{\prime };j^{\prime }\right),$ the following comparison is true $\rho_{b}\left(i-1;j-1\right)\geq \rho_{s}\left(i-2;j-1\right)\cup \rho_{s}\left(i;j-1\right)\cup \rho_{s}\left(i-1;j-2\right)\cup \rho_{s}\left(i-1;j\right)$, i.e., the number of same-species neighbor cells to (*i*;*j*) is superior or equal to that of one of its different species neighbor cells.

Reproducing biological systems’ complexity is not the goal, so to best approach the propagation mechanism of osteosarcoma, a probabilistic model of cell state change is introduced, the random element taking the place of the many biological parameters we cannot simulate. As such, the sum influence $\vec{v_{b}}\left(i;j\right)$ is normalized by its maximum value, before being compared to a randomly generated number R between 0 and 1, the transition of state being validated if R is inferior to the calculated value. Other parameters are introduced to manipulate the comparative probabilities between bone and sarcoma:

(6) $$R\leq \frac{\alpha_{bone}}{1-\beta_{bone}}\left(\frac{\vec{v_{b}}\left(i;j\right)}{{\vec{v_{b}}\left(i;j\right)}_{max}}-\beta_{bone}\right)$$

where the β parameter is an activation threshold and α is a comparative probability adjustment.

#### 2.9.2. Three-Dimensional Cellular Automaton

The first model of a cellular automaton is 2D, mainly for simplicity of modification and the lower number of calculations needed to run a simulation. The simulation represents the three species in white–gray–black: bone in white, sarcoma in gray, and healthy tissue in black.

A starting picture is transcribed in three Boolean matrices of the different species’ presence. Alongside this, matrices of normalized influence at a certain distance between bone and sarcoma are generated using the distance law chosen and the cut-off distance of effective influence.

The iterative process of the cellular automaton is then started, determining the cells with the possibility to change through contact and in agreement with the interface roughness rules. Influence matrices are then applied to the relevant species presence matrix and summed. That summed value is then compared to a randomly generated number, which, if inferior to the sum, activates the cell’s species change.

The three modified species’ presence matrices are then transcribed into the black–gray–white style of picture. Some of those pictures are assembled to form a movie.

Once the different parameters’ influence on form was qualified and a distance law was selected, the cellular automaton was transcribed in 3D. The laws and hypothesis remained the same, with the interface roughness laws adjusted to the different structure and number of neighbors of each cell.

The distance law was also adjusted, since the radial sum of influence depended on distance squared, whereas it was distance simple in 2D models, with a new propagation formula along the lines of $\vec{v_{b}}\left(i;j;k\right)=\vartheta \left(i;j;k\right){∭}_{V}\;f\left(d\right)\gamma_{V}\left(i^{\prime };j^{\prime };k^{\prime }\right)dV$.

## 3. Results

### 3.1. Early Stage of Osteosarcoma Development

In order to determine the first steps of the tumor propagation process, osteosarcoma cells were injected into mice through the tibial anterior muscle in close contact with the tibia periosteum [35]. Animals were sacrificed 7 and 13 days after tumor cell injection. At that time, mice did not show any sign of pathological behavior (no cachexia or frailty, which may appear around 15–20 days post-tumor cell inoculation). Control samples for bone studies were chosen as counter-lateral non-inoculated tibias. At the time of sacrifice, the limbs were cut off and frozen, before being sliced at 100 μm thickness, alternating support between nalophan and glass depending on the further characterization techniques, with some slices lost upon cutting.

#### 3.1.1. Optical Observation of Intracortical Bone Overproduction

**Figure 3:**
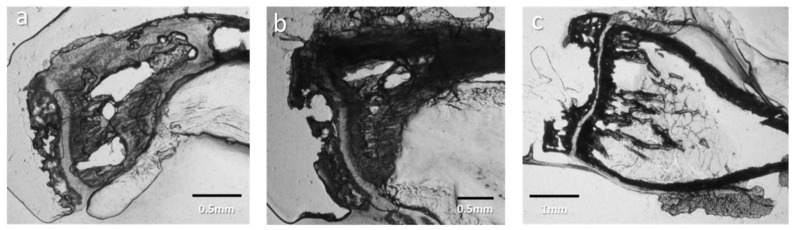
Slices of samples from inoculated mice tibia. (**a**,**b**) Two slices, D7.1 and D7.2, of the same 7-day-inoculated mouse tibia. (**c**) Slice D13 of the 13-day-inoculated mouse tibia.

The slices were first observed on the basis of experimental characterizations. A lack of cut-based degradation and size were the criteria in selecting the slices (Figure 1). This provided the first observations of de novo intracortical bone. Seven-day-inoculated slices (Figure 3a,b) display plentiful trabecular medullary bone, to the point where the porous character of the structure is hardly seen, with the bone appearing very dense at a 100 μm thickness. In contrast to controls, 7-day-tumor-bearing sample slices (Figure 1, first and third columns) show abundant trabecular bone in the metaphysis but are highly porous near the growth cartilage structure. Tibia slices from 13-day-inoculated mice (Figure 1 and Figure 3c) display a contrasting, nearly empty medullary cavity, with enough material left in a consistent fashion to discount damage by slicing as the exclusive cause.

It appears that the trabecular bone at diaphysis suffers from some changes between osteosarcoma samples and controls: 7-day samples present a potential increase in bone formation in comparison to control samples, whereas at day 13, samples from tumor-bearing mice display a medullary cavity that is near-empty in comparison to controls. Those observations are consistent in the successive slices from all the samples (Figure 1).

#### 3.1.2. Microfocus Small-Angle X-Ray Scattering of Bone Structure

**Figure 4:**
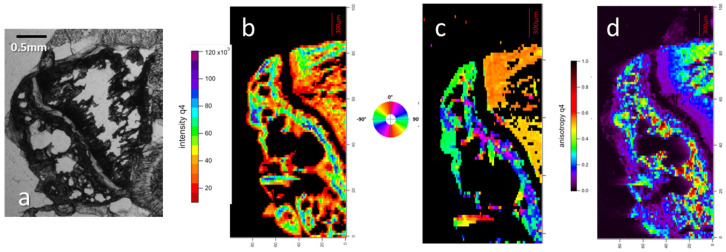
Control sample data. (**a**) Optic microscopy picture of control sample slice. (**b**) Maps of SAXS patterns’ analysis for intensity, orientation (**c**), and anisotropy (**d**), realized on apatite signal q ∈ 0.0423;0.250 Å^−1^. Intensity correlates with the amount of hydroxyapatite present, orientation is the direction of the diffracted signal, and anisotropy is a measure of the orientational organization.

**Figure 5:**
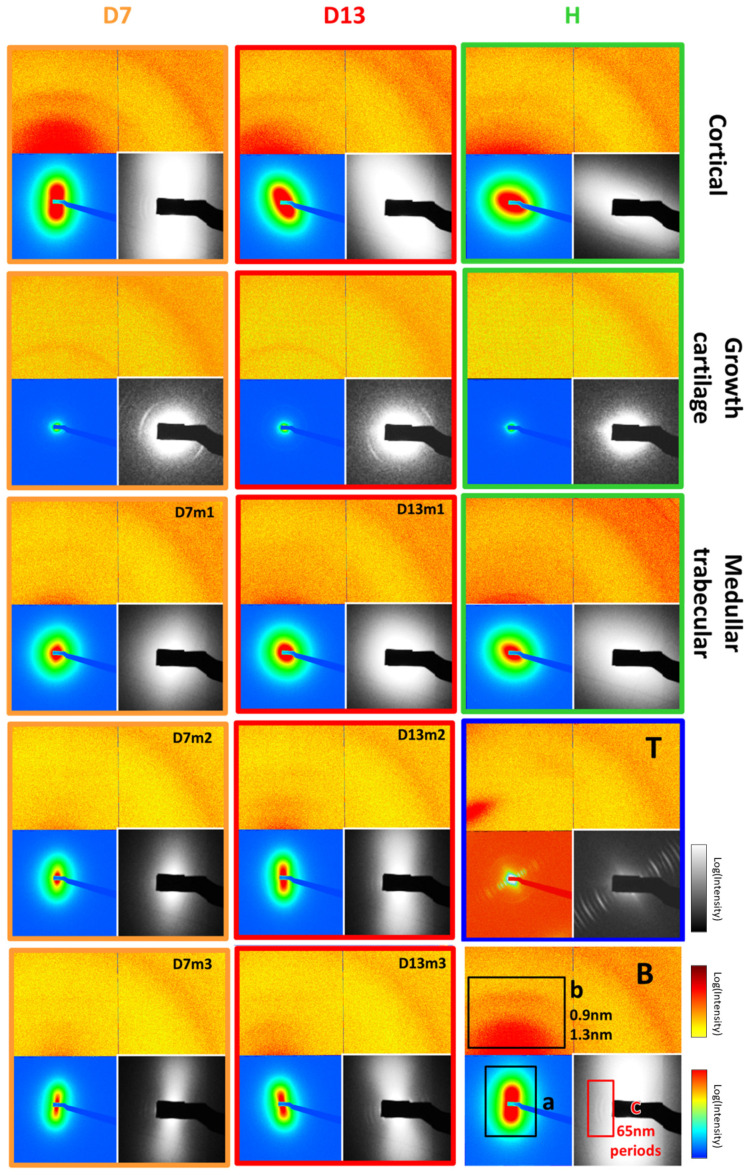
SAXS patterns of murine bone samples. B is the legend; the upper part is on a linear scale to display b, the lateral collagen packing, between 0.9 and 1.2 nm. We can observe, in this part, that the prevalence of the collagen packing signal and anisotropy depends on the type of bone material. Orientation is aligned with the apatite signal in the lower left part of B, which is in continuity with the upper part, albeit with the scale changed to logarithmic to better observe the diffusion signal from apatite crystals. In a, we can observe the intensity of the apatite signal, the anisotropy (degree of deformation from round which is without orientation), and the orientation of the apatite crystals at the given spot of the bone. The lower right part of B is also in a log scale with an 8× magnification to observe c, the collagen axial period signal, and how it appears clearer with greater distance from the growth cartilage (higher values of m in the figure). T is the tendon signal, with a strong type I collagen signal. Cortical D7, D13, and H are cortical bone from, respectively, the inoculated samples at 7 and 13 days and a healthy control sample. Growth cartilage is the growth cartilage of those samples. The rest m is the trabecular bone in the medullar cavity of said samples near the growth cartilage. The increasing number after m is the signal from further away in the medullar cavity of, respectively, the inoculated samples at 7 and 13 days.

Five representative samples were dedicated to the small-angle X-ray scattering (SAXS) mapping of typically 3 mm × 3 mm surfaces. This was performed with a 17 × 17 μm microfocus beam with a 30 μm step. Thus, 10,000 to 40,000 2D SAXS patterns were collected per slice. The most representative are depicted on Figure 5. The range of structure sizes covered by this device is from 6 Å to 300 Å (Figure 4).

For examples of 2D X-ray scattering spectra and examples of SAXS spectra of three sample slices, a healthy control (H) and 7-day-inoculated (D7) and 13-day-inoculated (D13) samples are presented (Figure 5). These were taken from cortical bone and growth cartilage, as well as at an increasing distance from the top of the proximal tibia, in the medullary cavity of trabecular bone.

Three zones of each spectrum picture represent different visualization adjustments, with the nomenclature being indicated in picture B. The bottom-left quadrant is the bone apatite mineral (HA) signal (a) around the incident beam, corresponding to a 12 to 100 Å range. The angular anisotropy of the signal indicates the global crystal orientation and quality. HA crystals are known to be aligned on the collagen fibrils scaffold [36], making their orientation fit with that of collagen lateral packing. The upper part showcases the lateral packing of the collagen signal (b) in continuity with the bottom-left part using different intensity scaling. The lateral packing of collagen is documented to be quasi-hexagonal, with a repetition distance around 13 Å [37]. The right half signal is due to sample support and ignored henceforth. The bottom-right quadrant showcases collagen 67 nm staggered (c) longitudinal packing signal. It is a 4× zoom of the bottom-left quadrant with specific intensity scaling, with the signal orthogonal to the lateral packing.

As an example, the dried tendon of the sample is nearly completely composed of type I collagen, and lacks any of the apatite signal of bone (Figure 5T). The figure showcases the signal from both the lateral (a) and longitudinal (c) packing of collagen. The pictures of Figure 5 cortical bone signal present no significant difference between samples, except for orientation (b), which is purely an effect of slice positioning. However, D7 and D13 display an interesting second arc of signal at the middle height of zone a, which appears to be consistent with the signal from the lateral packing of collagen, being in the same direction. Its presence in control (H), as far as we can tell, is unverifiable due to orientation making the signal position lie outside of the detector. No significant differences appear between the pictures of medullary trabecular bone (Figure 5(m1–m3)) at equivalent distances from growth cartilage. The observation of the trabecular bone area at increasing distances from the growth cartilage reveals an increase in the anisotropy of the HA signal, which allows for better observation of the collagen longitudinal packing (c) signal (Figure 5(D7, D13m1–m3)).

In Figure 5, corresponding to the growth cartilage of inoculated samples D7 and D13, the collagen packing signal is absent in the healthy control samples. This organized collagen is supposed to be absent from growth cartilage that is less organized. The signal also appears to be isotropic, unlike that of bone. The collagen SAXS signal also seems to present a secondary peak that we have not been able to find in the literature.

The full analysis of bone material by 2D SAXS pattern is highly challenging [18,37,38,39] and mainly requires absolute intensity data that were not accessible during the experiments.

Apatite crystal analysis manual fit proceeded as follows: The first approach was realized by fitting the 1D SAXS signal after radial integrations of 2D signals centered on the position of the incident beam. The procedure is classical in SAXS pattern analysis, but is time-consuming and may have fit or convergence difficulties. SASFIT [40] was used with a model of apatite rectangular parallelepiped nanocrystals form factor and a weak-interaction Percus–Yevick hard-sphere structure factor.

**Figure 6:**
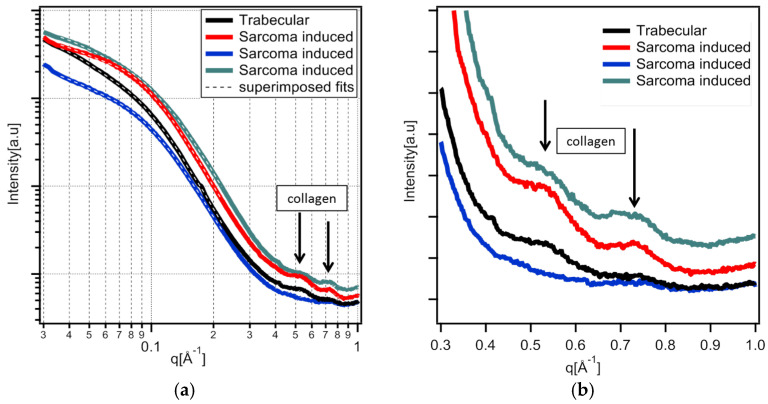
One-dimensional SAXS spectra of infected bone samples data. (**a**) Examples of radially integrated data of apatite signals from inoculated samples and the superimposed Sasfit fit. Trabecular bone from the 13-day-inoculated sample, and three different distances from growth cartilage sarcoma-induced bone-like material in the 7-day-inoculated diaphysis. (**b**) Zoom-in on the quasi-hexagonal peak of lateral collagen packing (q = 0.54 Å^−1^) and a secondary peak coherent with the first in presence, signal orientation, and anisotropy (q = 0.72 Å^−1^).

The variety of HA SAXS data are fit well, as demonstrated by the superimposed fit shown in Figure 6. Nanocrystals are needles with a thickness of 14–19 Å, a width of 34–40 Å, and a length more than 120 Å. A slight repulsive interaction of 4–10% between the needles is necessary to increase the fit quality.

It is noteworthy that mapping analysis of each SAXS spectra is not possible through SASFIT automatic fitting because of convergence problems. Thus, a new method has been developed to evaluate these parameters and generate corresponding maps of the packing collagen and the apatite structures.

#### 3.1.3. Microfocus SAXS Mapping of Bone Structure

**Figure 7:**
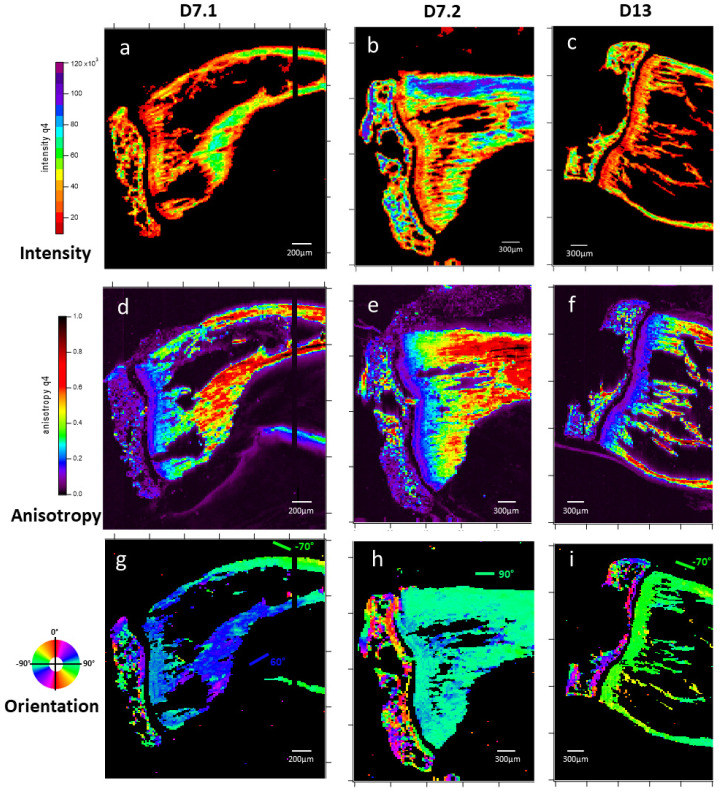
Maps of SAXS patterns analysis in terms of intensity (**a**–**c**), anisotropy (**d**–**f**), and orientation (**g**–**i**), realized at apatite signal q ∈ 0.0423;0.250 Å^−1^. Samples are two slices of 7-day-inoculated bone (D7.1 and D7.2) and a slice of 13-day-inoculated bone (D13). Each map results from the analysis of 40,000 2D X-ray scattering patterns. Intensity is an indicator of apatite presence and comparative quantities, with regions of interest being differences between trabecular and medullar bone, epiphysis and diaphysis, and the gradient into diaphysis from the growth cartilage. Apatite orientation is typically aligned with bone structure. One hope was to witness some decrease in organization correlating with osteosarcoma development. Here, we witness the homogeneity of diaphysis bone orientation versus the complex structure of epiphysis. The standard deviation in a 400-point region of diaphysis is >4°. Higher anisotropy values indicate a higher degree of orientation homogeneity, and here all samples display an increasing gradient in the diaphysis starting from the growth cartilage, displaying a dynamic of aging and apatite evolution in the diaphysis. A 350 data-point region in the higher values of map (**e**) gives an average of 0.76 for a 0.13 standard deviation.

The automated processing of raw 2D SAXS spectra requires some caution due to many available artifacts or possible erroneous measurements, such as detector saturation or excessively low intensity. Because bone SAXS mainly finds its origin in the inorganic crystal, the first processing was focused on HA crystal signals that range roughly from 25 to 150 Å. Indeed, at smaller angles (larger distances), some detector saturation is often observed. Signal intensity measurement of each acquisition is performed by summing intensity values of the signal in the HA range (Figure 7a–c). The most probable cause of disparities in the value range between mappings is the difference in slice thickness. A higher density of HA appears in the epiphysis (expanded end of the long bones) and cortical bone, with a lining out of the growth cartilage also present after a consistent thickness is seen in each slice. A total intensity of 104 counts is used as a threshold under which the presence of bone material cannot be confirmed, and pixels of the different mappings are set to 0 using this parameter.

Inoculated sample slice mappings of apatite were obtained: The quality of the orientation (Figure 7d–f) and the orientation itself (Figure 7g–i) of the SAXS were then mapped. The epiphysis slices present an expectedly intricate network, with different orientations (Figure 7g–i) fitting trabecular bone built to distribute forces exerted over the superior part of the bone. The diaphysis displays a near-homogeneous orientation along the bone direction, with variations contained within a ±15° span, which seems more surprising for the trabecular part [41]. HA orientation anisotropy further differentiates the epiphysis from the diaphysis, with the former being generally near isotropic at an average sample thickness of ~100 μm, except for in some highly aligned spots (Figure 7d–f). Diaphysis displays near-homogeneous anisotropic characteristics, which are correlated with the distance from the growth cartilage in a positive gradient.

**Figure 8:**
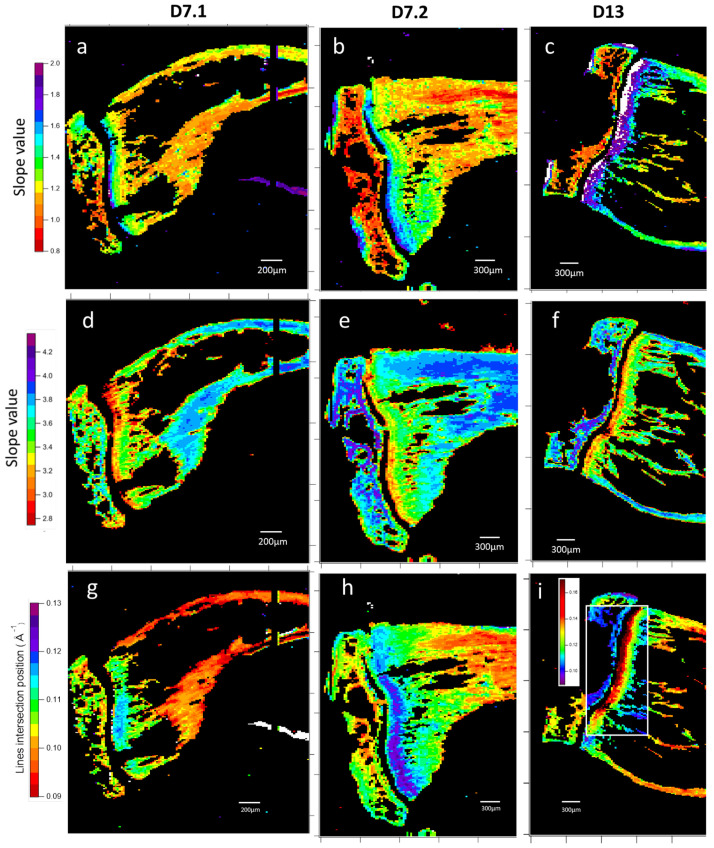
Maps of SAXS 1D spectra analysis with first slope value (**a**–**c**), second slope value (**d**–**f**), and line fit intersection position (**g**–**i**) of the bone apatite crystals’ signal. Samples are two slices of 7-day-inoculated bone (D7.1 and D7.2) and a slice of 13-day-inoculated bone (D13). Slopes are determined by the log/log linear fit of the 1D SAXS signal in the q ∈ 0.042;0.073 Å^−1^ range for the first slope and the q ∈ 0.150;0.250 Å^−1^ range for the second. The line intersection position is calculated from fits values. Each map results from the analysis of 40,000 2D X-ray scattering patterns. The first slope value is in the Fourier zone and refers to apatite crystals’ shape, with values of 2 being platelets and values of 1 being needles, which indicates, in the diaphysis gradient of the growth cartilages, an evolution towards what could be considered needles for mature bone, like in the epiphysis, although far less so in the 13-days sample. The average of 300 data points in (**b**) the rightmost part is 1.18, with a standard deviation of 0.14. The second slope is for the Porod zone, with values of 3 being a rough interface for apatite, and 4 being smooth. In addition to the shape evolution in the bone, we have a gradient indicating a smoothing of the apatite interface across samples. In the same zone as previously, the average value is 3.85 for a standard deviation of 0.04. Slopes’ intersection q position correlates with crystal perimeter and displays another diaphysis evolution of apatite with a perimeter gradient increase.

The 1D radially integrated SAXS signal of apatite nanocrystals was then analyzed as previously described. Data at both very low and intermediate angles were analyzed by linear regression of the log–log plot. The slopes are related to the power law exponent η and may give some insight into the shape (Figure 8a–c) and the rugosity (Figure 8d–f). Most interestingly, the HA crystal perimeter (Figure 8g–i), which is equivalent to parallelepiped crystal dimensions of 2 (*a* + *b*), can be precisely estimated and mapped. Both D7 sarcoma inoculated samples exhibit a positive gradient in the diaphysis, with a lower value in the middle of the medullary cavity’s near metaphysis (Figure 8g,h). Perimeter measurements above 90 Å (red), up to over 160 Å (white), appear near the growth cartilage and in external spots of the epiphysis, suggesting the possibility of the measurement of something different. Besides those particular points, the upper limit of apatite crystals perimeter seems to be 140 Å (purple). These perimeter variations in apatite crystal are the first indications of a gradient of ultra-structured bone excess induced by the tumor cells. We find a gradient of anisotropy in the same region, suggesting an impact from apatite crystal size evolution.

**Figure 9:**
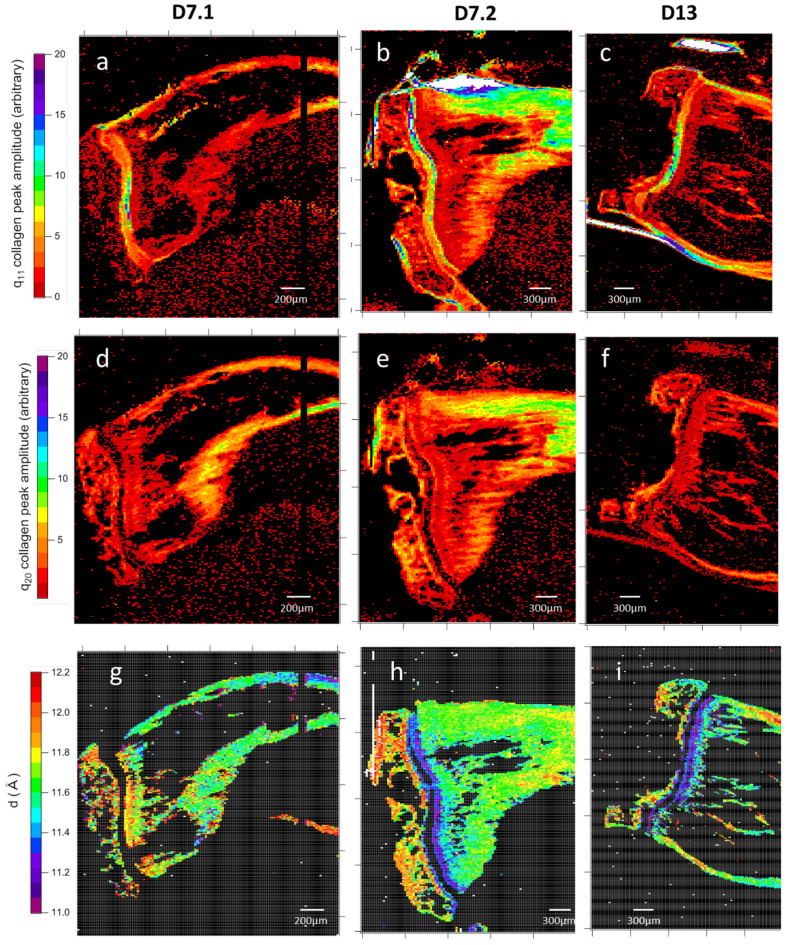
Mappings of collagen lateral packing peaks’ intensity and calculated crystal cell size. The amplitude of the peaks of collagen lateral packing (**a**–**f**) and quasi-constant interdistance (d in Figure 10) (**g**–**i**) size. Lateral packing peaks are on q ranges of 0.51–0.59 Å^−1^ and 0.685–0.765 Å^−1^ and cell sizes are calculated from their respective indexations as (11) and (20) planes. Samples are two slices of 7-day-inoculated bone (D7.1 and D7.2) and a slice of 13-day-inoculated bone (D13). Each map results from the analysis of 40,000 2D X-ray scattering patterns. The q_11_ orientation is very present in cartilages and sinew (white saturated parts), with higher values besides those correlating with high intensity values (Figure 7a–c). The q_20_ orientation peak is for its part exclusive to bone and correlated with the first. The calculated crystalline cell size is near homogeneous in post-growth cartilage diaphysis, with an average of 11.68 Å with a standard deviation of 0.13 Å over 3500 data points along the length of sample D7.2.

Sample slice mapping of collagen: Collagen packing generates two broad scatterings around 0.5 Å^−1^ and 0.7 Å^−1^ (Figure 6b). Despite the very low intensity of this, as observed in Figure 5, both peaks have been observed in all samples (Figure 9a–f). In most collagen tissues, the 0.5 Å^−1^ peak appears in bone and tendon, tissues which are constructed of organized type I collagen. It also appears in the growth cartilage of inoculated samples and not the healthy ones that display the expected result. The amplitude is comparable to that of dense cortical bone. The origin of the 0.7 Å^−1^ peaks remains unclear in the literature. For instance, it has been observed and associated with crystalline apatite signals [41]. This attribution is incompatible with the observation of a 2D signal that systematically exhibits the collagen packing orientation. From our later observation of apatite crystalline peaks in wide-angle X-ray scattering (WAXS), we consider that peak to be a secondary signal from collagen lateral packing. This second peak of collagen lateral packing is observable exclusively in bone material and presents no amplitude peculiarities (Figure 9a–f). The two peaks of collagen lateral packing coexistence suggest the existence of two different distances in the packing. The admitted packing of collagen in bone material and tendon is quasi-hexagonal, a near-crystalline structure in the transversal plane. The first two crystallographic peaks for a distorted hexagonal structure are the (1;1) and (2;0) planes of a centered rectangular unit cell. The calculated elementary cell size for the two different possible indexations result in only one coherent result, with the first peak as the (1;1) reticular plane and the second as the (2;0) one.

**Figure 10:**
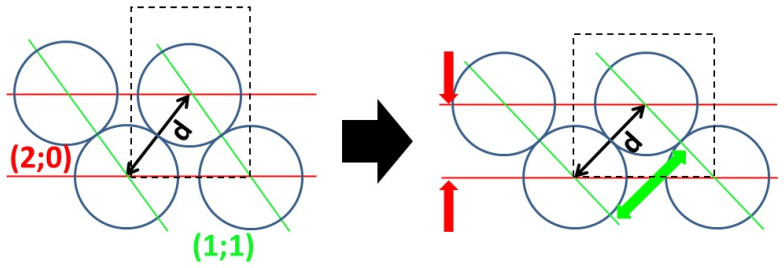
Collagen interdistances shifting via the anisotropic deformation of a rectangular sub-lattice (hyphen black), with a constant interdistance (d). The vales of (2;0) and (1;1) are the smallest-angle diffuse scatterings of this collagen packing.

**Figure 11:**
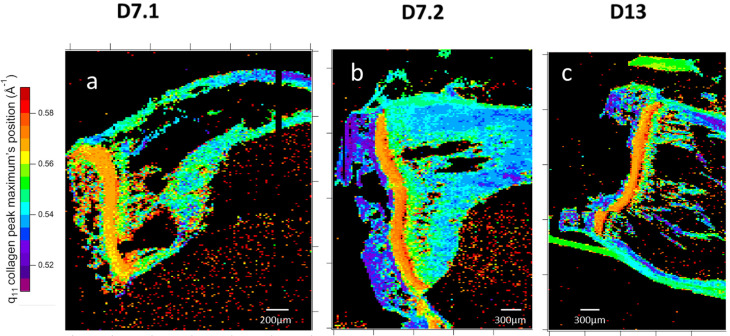

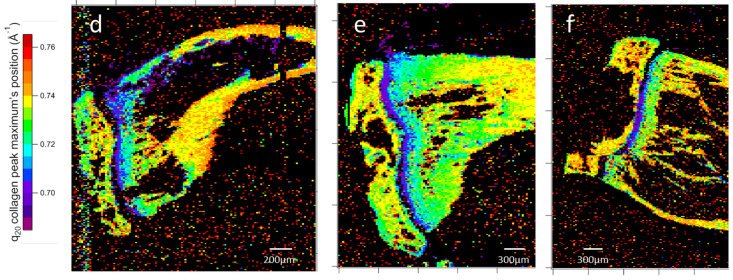
Mappings of collagen lateral packing peaks’ maximum position. Lateral packing peaks are in the q ranges of 0.51–0.59 Å^−1^ of the (11) plane and of 0.685–0.765 Å^−1^ of the (20) plane. Samples caption are two slices of 7-day-inoculated bone, D7.1 (**a**,**d**) and D7.2 (**b**,**e**) and a slice of 13-day-inoculated bone, D13 (**c**,**f**). Each map results from the analysis of 40,000 2D X-ray scattering patterns. The evolution of peaks in q position values in the diaphysis correlates with all the previously seen gradients and seems to indicate a shift in collagen packing fitting with apatite crystal shape, size, and interface.

One diagonal of this distorted hexagonal cell is constant (Figure 10), and the close contact distances between collagen molecules can be mapped (Figure 11). Hence, the lateral packing in the growth cartilage is at 11.6 ± 0.3 Å, tighter than anywhere else in the samples. In the diaphysis, variations in collagen peaks indicate a spacing gradient (Figure 9g–i) of 0.6 Å. Epiphysis values are similar to those of far diaphysis. Epiphysis displays mostly homogeneous spacing between collagens, and does so at a higher value than far off diaphysis. The apparent lack of collagen in the near-metaphysis medullary cavity is actually due to a combination of low anisotropy and the coverage at this scattering angle being about a fourth of the total signal.

#### 3.1.4. Wide-Angle X-Ray Scattering of Overproduced Hydroxy-Apatite

**Figure 12:**
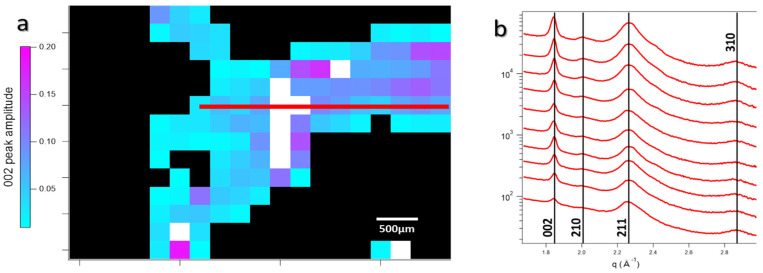
(**a**) Maps obtained from the 256 WAXS pattern of D7.2, evaluated from the crystalline apatite 002 peak amplitude. (**b**) Staggered WAXS spectra along the red line in (**a**), with the classical indexation of apatite crystalline peaks. No signal associated with sarcomatic bone apatite can be witnessed in these measures at such early stages.

The shape of HA nanoplatelets has been thoroughly analyzed. In order to have them fully characterized, the crystallinity was studied by wide-angle X-ray scattering (WAXS) on a more intact sample (D7.2). Mappings of the most intense crystalline peaks of HA, 002 (3.4 Å) and 211 (2.8 Å) [42], were realized in house, with a step between acquisitions of 250 μm. Diaphysis bone HA displays several crystalline HA diffraction peaks (Figure 12), without any significant differences between healthy and tumor-induced samples at early stages, unlike in advanced osteosarcoma [43].

#### 3.1.5. Mechanical Properties

**Figure 13:**
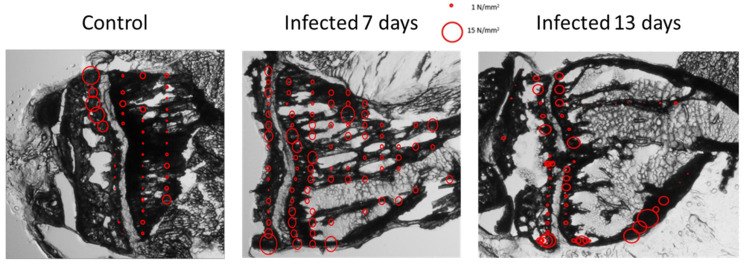
Micro-indentation Marten hardness maps on healthy control sample and inoculated samples at 7 and 13 days. Red circles diameter is proportional to the measured hardness value obtained at the site of the circle center.

The macroscopic quality of the bone-like material ultra-structure was further characterized by means of a depth-sensing micro-indentation technique. The obtained Martens hardness measurements provide some insight into the spatial distribution of the mechanical properties. The epiphyseal area hardness was found to greatly fluctuate along the growth cartilage in all samples, from about 1 N/mm^2^ in the interior to around 10 N/mm^2^ (Figure 13).

The diaphysal bone hardness in the healthy control sample is quite low on average in comparison to the values measured on inoculated samples, despite the fact that a maximum value of 4N/mm^2^ is incidentally obtained in some indentation sites. Inoculated samples of diaphysis are typically significantly harder, with a maximum value of about 8 and 10 N/mm^2^ 7 days and 13 days after inoculation, respectively.

#### 3.1.6. Cell Identification by Microscopies of Overproduced Bone-like Materials

In the light of potential overproduced bone material, we strive to check for any difference in the presence of osteocytes in the structure or osteoblastic lining. Since most of the SAXS (more than 40,000 spectra) were similar in the intra-trabecular area of D7, we assume that all parts of the samples exhibit the same feature, allowing us to investigate only one well-characterized part of the bone-like material. Back-Scattering Electron (BSE) observation required thinner slicing, which was from a hundred microns to a few microns.

Scanning Electron Microscopy (SEM) observations were performed using BSE mode, which is equivalent to Energy-Dispersive Spectroscopy (EDS). The microscopy of bone exhibits intensity proportional to the amount of mineralization. Quantitative analysis of calcium and phosphate was performed by EDS at 4 different sites along the diaphysis of D7 (Figure 2a) and Ca/P ratios are depicted in the bottom-left picture’s table. In healthy samples, the mean ratio value is between 1.5 and 2 [44,45], as observed here in the sarcoma inoculated sample (Figure 2a).

Osteocyte: We sought to check the nature of the apparent surplus bone-like material in 7-day-inoculated samples pointed via optical microscopy observation. All the relevant elements of regular trabecular bone are observable, including the osteocytes inside the bone structure (Figure 2c). The osteocyte distribution is typical of bone tissue, indicating controlled bone growth, and consequently strongly supports the proposition that studied bone-like material has all the characteristics of bone materials.

### 3.2. Topology at Late Stage of Osteosarcoma Development

The macroscopic morphology of late-stage osteosarcoma (28 days) provided a basis for what to reproduce in the topology through simulation in the cellular automaton. Indeed, to verify our hypothesis, such modeling must be able to generate, in silico, the final-stage morphology of osteosarcoma.

#### 3.2.1. Micro-CT Reconstructions

**Figure 14:**
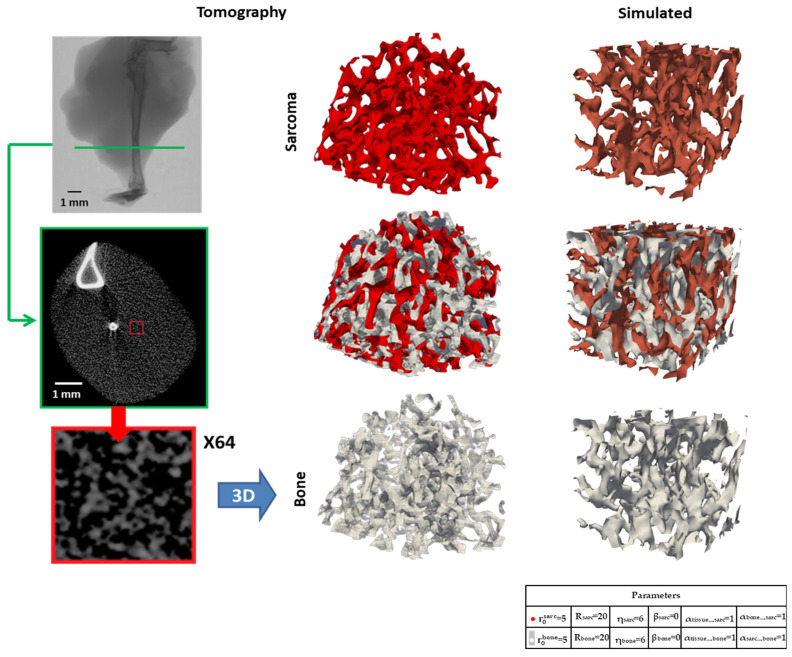
Left: osteosarcoma mouse tibia MicroCT (**top**) with a slice example (green in the **middle**) and a zoom (**bottom**). Center: 3D surface reconstruction from MicroCT data of the bone topology (white: high density) and sarcoma cells (low density: red). Right: 3D simulation model with the same color code. The left parts are the template from which the 3D surface reconstruction were performed by using certain signal levels cut-offs to separate bone and sarcoma, providing a reference structure to analyze in comparison to simulated structures. FFT were used to determine some underlying tendencies in the measured tomography structure and in an attempt to quantify the level of disorder the simulation must reach.

We used Micro-CT slices of osteosarcoma bone [25] to build 3D models of the osteoid and tumoral phases (Figure 14 and Video S1). The biological system has been thoroughly characterized in previous studies [25]. The signal perceived through this method, presented in white in the slices, corresponds to the presence of bone-like material. We screened every slice’s pixel value, ascribing high electron density values to the presence of osteoid (white in 3D representation), and low values to the presence of sarcoma cells (red in 3D representation). A gap between the two values is kept for uncertainty as well as for the readability of the interlocked 3D models produced (Figure 14 and Video S1). The analysis of those models presents the fact that no isolated part of the osteoid or sarcoma occurs, with all elements of the same nature being interconnected. The overall structure of each phase presents many holes, in a sponge-like manner, with a lot of generated surfaces, possibly favoring exchanges between the two.

The estimated whole is considered as a bi-continuous structure. Using these reconstructed 3D models, we applied Power Spectrum analysis to determine if there were any characteristic distances expressed in the osteoid structure that correlated with the overall structure of osteosarcoma. No direction allowed for the expression of a clear spatial frequency or the object repetition direction of the osteoid structure. The most actionable information is a distribution set of expressed frequencies, with a level of disorder in the structure that would act as our guideline in reproducing the topology of osteosarcoma through simulation.

#### 3.2.2. Two-Dimensional Topology Modeling

The morphology of the trabecular bones was extensively numerically simulated in order to understand the mechano-transduction mechanism [46,47]. This approach has been successful in demonstrating the osteocyte sensor function in the remodeling process and the 3D outstanding topology of intracortical bone. We developed a 2D then 3D numerical simulation by replacing the usual mechanical negative feedback with a tumor cell positive feedback, as observed in the first part of this paper. We extended a cellular automaton [48] with the aim of reproducing the topology of osteosarcoma’s osteoid structure, the logic being that a set of rules able to reproduce the observed topology would be a possible approximation of the actual rules of osteosarcoma propagation and mechanisms. Results tend to greatly resemble those of more complex models, showing peripheral pattern formation simulation of vascular mesenchymal cells’ self-assembly [49,50].

**Figure 15:**
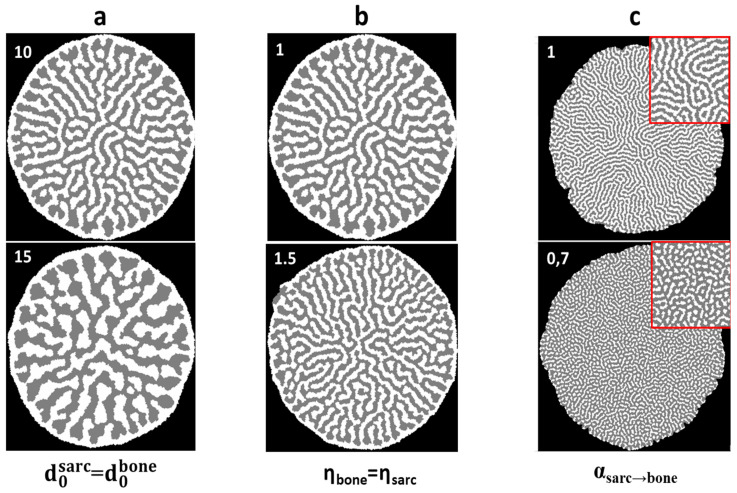
Simulation results sample. Comparison of simulation results with variation in one or two parameters. (**a**) d0—maximum influence distance, affecting structure characteristic sizes and shape disparity. (**b**) η—distant influence ratio ponderation, affecting structures characteristic sizes and connectivity levels. (**c**) α—species propagation speed comparative ratio, affecting the presence ratio and connectivity levels.

The parameter optimization of the 2D model: The model is built up around a synergistically long-range influence between bone synthesis and tumor cell growth. The parameters are those defining the distance law of influence, and the probabilities threshold, described in Equations (5) and (6). By modulating these parameters, we sought to get ever closer to a model of osteosarcoma topology. We present, in Figure 15, the impact of our parameters on topology at the end of simulation (3000 iterative loops).

The structure size is affected by the distance of influence parameter d0. Higher values produce bigger structures and greater shape heterogeneity. The parameter of the law of distant influence maximum effect intensity η negatively affects structure size by favoring the d0 value more heavily, but favors connectivity, with less isolated elements. The corrective probability parameter α affects species propagation speed: the greater the difference in the comparison values, the more higher-value species will be present. Those differing ratios express themselves in the topology by reducing the connectivity of the slowest propagating species, going from near-continuous phases at equilibrium or superior speeds to separate blocks. The probability threshold β possesses a similar effect: the higher the value, the slower the propagation speed of the associated species, with the same consequences as for α.

**Figure 16:**
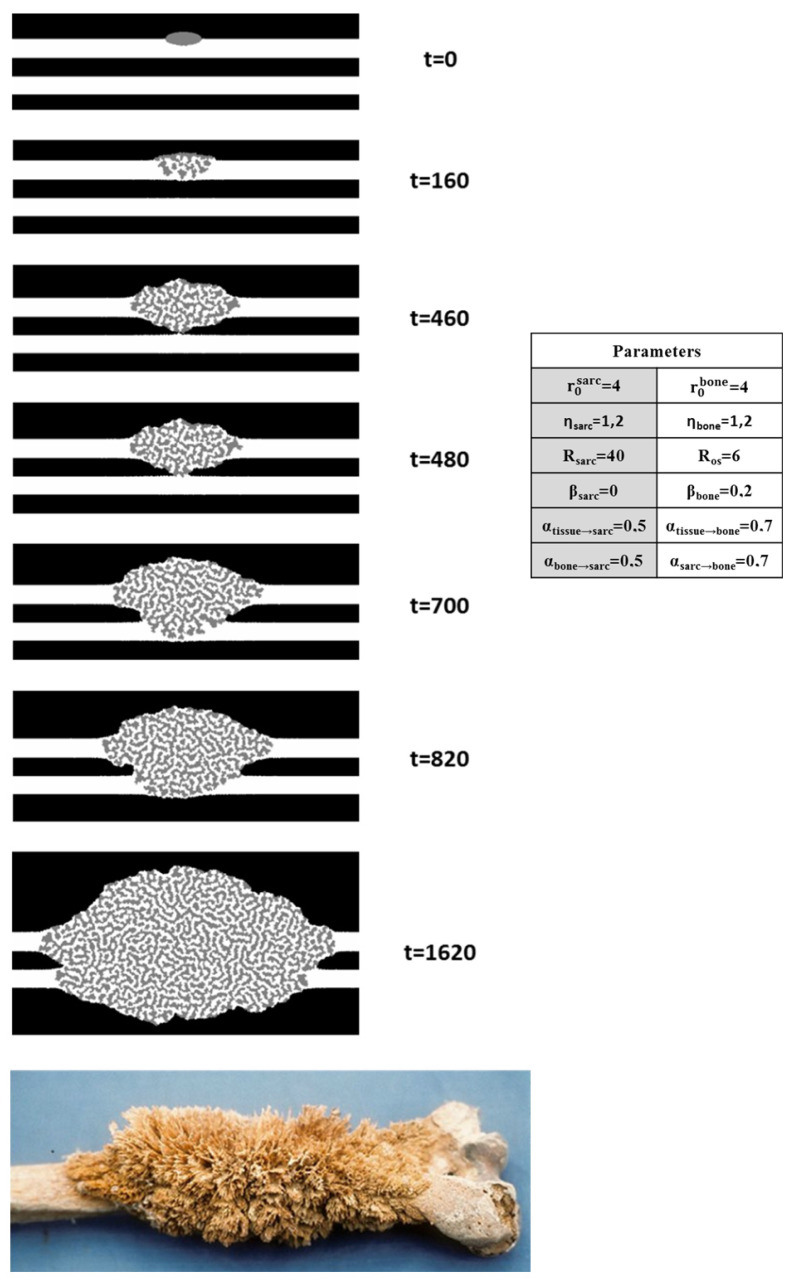
The evolution of a simulation at t iterations, with a set of parameters (right) that result in our best approximation of osteosarcoma’s disordered sponge-like topology. A picture of an osteosarcomatous human bone [35] (**bottom**) is given for reference [51]. The simulated bones are represented in white and the sarcomatic flesh is shown in gray, with black here used as an inert environment. The mechanism of propagation is based on a positive cross-catalysis process.

Initial morphology influence: Sets of different initial morphologies were used to ascertain their impact on the development of our simulated model of osteosarcoma. The initial morphology controls the direction, shape, and speed of the propagation of bone and sarcoma in our model, with the determining instant of contact speeding up the process greatly. The interface with healthy tissue is also conditioned by the prevalent species on the side of the contact with the parameters in use. An example of the progression of a simulation with one of the sets considered among our best is also included (Figure 16 and Video S2).

This model displays the quick degradation of existing simulated bone (osteolysis), with propagation favored by pre-existing bone, and a slower lateral growth of a mix between bone and sarcoma structures.

#### 3.2.3. Three-Dimensional Topology Modeling

The 2D model presents some limitations, the more obvious one being that it is impossible to reproduce a slice of a bi-continuous structure through the evolution of a 2D model, leading to the development of a 3D model of cellular automaton. The rules and laws were adapted to a three-dimensional model, allowing for interaction between voxels. A preliminary exploitation of this model was applied to the determination of the set of parameters that would best fit the osteosarcoma structure reproduction in 3D.

Interestingly, the bi-continuous nature of the structure is faithfully reproduced, with characteristic sizes approached (Figure 14 right). However, the topology still remains too smooth and ratios too balanced compared to the tomographic results of osteosarcoma. We are very hopeful that our working hypotheses are sufficient to simulate and explain the sponge-like osteosarcoma morphology.

## 4. Discussion

The results presented in the manuscript give new insights into bone development during osteosarcoma growth. At a very short time of tumor development (D7), the ultra-structural methodology allows us to characterize the surplus bone features and their modulation within the medullary cavity. The concomitant growth cartilage evolution opens the way to propose an activation mechanism. The latter time observations at D13, completing the osteosarcoma vicious cycle, are instructive for proposing a minimal model explaining the osteosarcoma final morphology.

### 4.1. Bone-like Material Surplus at D7

#### 4.1.1. Structure of the Bone-like Material Surplus at D7

Bone generated during osteosarcoma growth is known as “osteoid” without having more details on the ultra-structure of the collagen–HA composite properties. Here, we show that 7-day-inoculated trabecular bone-like material is similar to cortical bone by X-ray scattering. Hence, collagen fibrillation and packing are conserved. Hydroxyapatite (HA) exhibits a classical nanorod shape and the WAXS signal indicates a crystalline state, exhibiting the feature of mature bone [52].

Orientation in the diaphysis remains homogeneous to the bone direction, with no anisotropy discrepancies. Moreover, mechanical properties are comparable in the medullary and the cortical bone areas on D7. All these results, combined with the presence of osteocytes in typical areas of this de novo bone-like material (Figure 2), confirmed that excess bone at D7 has the feature of healthy bone material.

#### 4.1.2. Structural Gradient Within the Medullary Cavity at D7

In the slices of 7-day-inoculated tibias, several structural gradients were observed in the medullary cavity from the growth cartilage to the inoculation site. HA crystals become more oriented, with a shape evolving from small platelets towards rods and a growing perimeter. Collagen packing signals evolve in the same direction, with a compression along the (2,0) plane suggesting an absolute triple-helix collagen spacing reduction. We expect that HA crystals growth will exert pressure on the collagen structure, packing it lower to accommodate it. All the observed gradients into the medullary cavity occur in the same direction and are in agreement with more aged bone being close to the inoculation site than next to the growth cartilage.

#### 4.1.3. Structural Evolution Induced by the Tumor in the Growth Cartilage

Osteosarcomas are often observed in growing adolescents, and we focus our interest on growth cartilage modification at the same time when bone surplus is observed at D7. Organized collagen in the growth cartilage is absent in healthy samples, whereas all inoculated samples display lateral and longitudinal packing of collagen signals. Thus, SAXS signals of collagen packing in the growth cartilage may be obvious signs of osteosarcomaactivity. Moreover, the BSE observation of healthy and inoculated samples required thinner slicing, with a reduction from a hundred microns to a few microns. These cuttings resulted in the slices from healthy mice being much more complete, suggesting that the improved hardness to piercing in 7-day-inoculated mice does not translate to shearing hardness. SEM observations of growth cartilage seem to indicate earlier mineralization in endochondral ossification, which is possibly linked to the presence of organized collagen. These differences in the structure suggest that osteosarcoma cells induce, at long distance, a significant alteration in the growth cartilage, which is mainly composed of chondrocytes.

### 4.2. De Novo Bone Synthesis Activation Mechanism

The main unexpected result of this study is the large bone material excess generated intracortically at a long distance of the tumor cell extracortical implant. The corresponding activation mechanism is unknown, but is expected to play a major role in osteosarcoma development and may involve a secreted partner. The bone material surplus quality is similar to that of cortical bone, suggesting that it was produced classically by osteoblasts. This is confirmed by the presence of osteocytes, which are trapped after mineralization. The osteoblasts are thus very quickly activated and necessarily migrate there from either differentiated or stem cell tissues. The huge amount of intramedullary bone-like material after a few days is in contradiction with the slow differentiation of stem cells into osteoblasts before the bone synthesis. This suggests either the pre-existence of osteoblasts in endostea or the existence of pre-differentiated cells able to quickly transform into osteoblasts. The growth cartilage degradation at D7 sheds light on an unexpected pathway involving chondrocytes that are the predominant cells. Indeed, chondrocytes are known to evolve toward osteoblastic cells in some endochondral stress contexts [53]. Moreover, osteoblasts are known to migrate over long distances under phosphate gradients [54].

**Figure 17:**
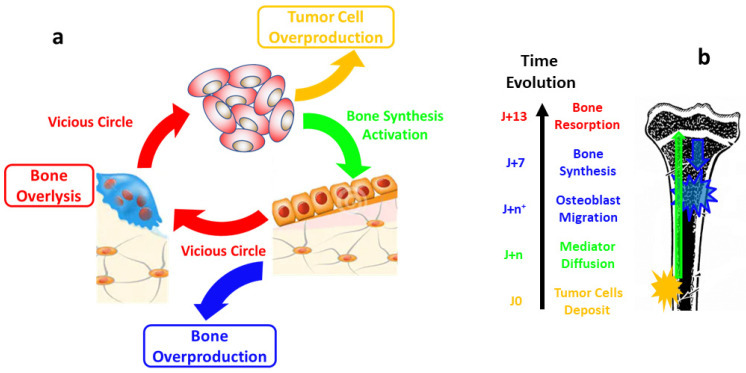
(**a**) The proposed cycle of tumor development by synergy between bone growth and tumor cell proliferation, in agreement with all observations and simulation. (**b**) The proposed early step of osteosarcoma development in the mouse model after extracortical cancer cell inoculation.

Both results together suggest a two-step activation mechanism (Figure 17b). Firstly, an unidentified mediator quickly diffuses from the inoculated tumor cells through the cortical bone and over a longer distance in the medullary cavity in order to activate the chondrocyte/osteoblast transformation. Secondly, another mediator, or the same, activates the fast migration of osteoblasts from the growth cartilage to the medullary cavity to produce the bone-like surplus. Such a long-range source of bone growth activation is in agreement with the bone ultra-structural gradient observed by SAXS in both apatite shape and collagen packing.

### 4.3. Bone-like Material Degradation at D13

The second unexpected result is the diminution of bone-like material, observed at D13 in the intramedullary cavity in more than 20 slices of tibias of tumor-bearing mice. This reveals that D7 de novo intracortical bone is damaged by a biological process until its disappearance or disappears through very bad mechanical quality and a deterioration of these parts of slices during the cutting. Notably, the D7 bone is in a large excess and consequently under mechanical stress, and it is filled with osteocytes. Under mechanical stress, osteocytes are known to activate bone degradation by recruiting osteoclasts [55]. This would explain the sequential order between D7 and D13 samples. In this proposition, the intracortical bone degradation is not related to the vicious cycle of osteoclast activation [56], but to the large excess of synthesized bone, generating huge compressive mechanical forces.

### 4.4. Sponge-Morphology

#### 4.4.1. Osteosarcoma Morphology Model with a Minimalist Model

The sponge-like morphology of osteosarcoma (Figure 14) is unusual in the cancer field. In general, tumor invasion is a single process, with only tumor cell divisions simultaneous to blood vessel development. The distinctive feature of osteosarcoma is the spatial and temporal combination of two growing processes of bone and tumor. Such concomitant processes arise most of the time from interconnected activations [20,21]. We propose a minimal model based on (i) well-known tumor cell activation by bone [17] and present (ii) our observation demonstrating the long-range activation of bone material synthesis by tumor cells. Both activations correspond to positive feedback loops in a very simple network (Figure 17a). The automaton simulations are only built up with these two approaches and result in very good morphological agreement (Figure 14). One major point in our simulations is the long-distance impact, which suggests the presence of a mediator with high mobility. Noteworthy, although the agreement is not final proof of the synergy, the lack of alternative explanation and the simplicity of the model makes it very likely that this is the case.

A cross-catalysis coupling has already been observed in the biomineralization processes of synthetic self-assemblies [2]. In this first example study, the aligned shape was controlled by the macroscopic chemical gradient as expected here during the early-stage study. In the absence of a macroscopic anisotropic gradient, a lack of macroscopic orientation is observed, but the alternation between bone and tumor tissues is the main feature. In both examples, even if the characteristic size is 105 larger, the growths of both inorganic materials and chemical/tissue template are hugely entangled.

#### 4.4.2. Biological Steps Included in Each Automaton Step

The osteosarcoma morphology can thus be explained by a synergy scheme (Figure 17a), in which tumor cells induce bone production, which in turn activates sarcoma proliferation. Both steps of this simplistic view will have to be clarified in the future. Tumor growth activation is quite well understood in the literature since bone excess participates in the vicious cycle [16,56], including osteoclast activity, which produces a mediator able to activate the tumor growth. On the contrary, we observed bone-like material production activated at long range by tumor tissue. This is rarely discussed in the literature. Tumor activation by bone tissue is suspected in some prostate cancers with simultaneous bone production by osteoblast [57,58]. It could also explain the presence of bone calcification in the pulmonary metastasis of osteosarcoma [59]. Early-stage investigations and previous discussions are in favor of two activation processes in which osteoblast activation could explain the large excess of bone observed in late-stage osteosarcoma. The mediators could be studied and identified using three-dimensional osteosarcoma models developed for advancing drug discovery [60].

This opens the question of the origin of the cell that synthesizes bone-like material in the case of osteosarcoma. In the literature, it is accepted, but not proved, that osteosarcoma cells can generate immature bone, which may result from the deviation of osteoblastic precursors from their “normal” differentiation into osteoblasts towards transformed cells. Here, we propose that activation of healthy osteoblast by tumor cells may explain the osteosynthesis observed in patients. We strongly believe that osteoblasts originate in the promotion of differentiation of perichondrial mesenchymal stem cell into osteoblast as observed in the osteogenesis controlled by YAP and TAZ [61].

## 5. Conclusions

In this paper, we enlighten the origin of the osteosarcoma sponge-like morphology by identifying the synergy between two entangled production steps: (i) bone-like material production activated by tumor cells and (ii) tumor cell activation by bone cells. In order to isolate the former step, we broke up the interlacing by investigating the early stage of osteosarcoma invasion at a very short time (D7) after the periosteum injection of osteosarcoma cells. This mice model is used in order to investigate, in depth, the early stages of osteosarcoma development. The observation of bone over-synthesis inside the medulla in contact with the metaphysis and marked growth cartilage degradation suggest an unexpected long-distance bone synthesis activation, whose mediator remains to be identified. The second step is associated with the vicious cycle between tumor cell development and bone degradation. Indeed, osteoblasts in close contact with bone produce cytokines and growth factors are known to activate tumor proliferation. This mutual coupling produces a rare synergy that explain the final osteosarcoma morphologies well: a 3D cellular automaton with this simple positive synergy accurately reproduces the bi-continuous sponge macroscopic structure that was analyzed from mice tumor micro-CT. Macroscopically, osteosarcoma can be understood as a symbiotic expansion of tumor and bone, generating a sponge phase mix of tumor cells and bone material.

From a fundamental point of view, bone tissues are biological systems that maintain both architectural and biological steady states by a network of varied feedback processes. Cancer development is often related to vulnerability in such a network. In osteosarcoma, the bone remodeling network is hijacked by the tumor to free space in healthy tissue by bone growth. The mechanical pressure required to develop is consequently applied by bone tissue instead of tumor cells, which are less stiff. This mechanism explains osteosarcoma’s aggressiveness very well. Additionally, the proposed osteosarcoma growth model offers new opportunities to identify innovative drugs by exploring the bone activation mechanism and mediators that can be applied to other bone pathologies linked to osteo-formation, such as bone metastases from prostate cancer [62].

## Data Availability

The original contributions presented in this study are included in the article/Supplementary Material. Further inquiries can be directed to the corresponding author.

## Supplementary Materials

The supporting information can be downloaded at: www.mdpi.com/article/10.3390/nano15050374/s1, Video S1. Rotating reconstructed from microCT structures. Video S2. Simulation of osteosarcoma structure propagation.
