# Supplementary material for: Sponge Morphology of Osteosarcoma Finds Origin in Synergy Between Bone Synthesis and Tumor Growth
Source: Nanomaterials (Basel). 2025 Feb 28;15(5):374. doi: 10.3390/nano15050374 (PMC11901559; doi:10.3390/nano15050374)
Supplement: Supplementary file 1 [file nanomaterials-15-00374-s001.zip › nanomaterials-3423286-supplementary.pdf]

Supporting Videos

Sponge morphology of osteosarcoma finds origin in synergy between bone synthesis and tumour growth

*Arnaud Bardouil, Thomas Bizien, Jérome Amiaud, Alain Fautrel, Séverine Battaglia, Iman Almarouk, Tanguy Rouxel, Pascal Panizza, Javier Perez, Arndt Last, Chakib Djediat, Elora Bessot, Nadine Nassif, Françoise Rédini and Franck Artzner**

Video S1. Rotating reconstructed from microCT structures with margin of sarcoma, sarcoma interwoven with bone and bone.

Parameters	
$r_0^{sarc}=4$	$r_0^{bone}=4$
$\eta_{sarc}=1.6$	$\eta_{bone}=1.6$
$R_{sarc}=100$	$R_{bone}=100$
$\beta_{sarc}=0$	$\beta_{bone}=0$
$\alpha_{tissue\rightarrow sarc}=1$	$\alpha_{tissue\rightarrow bone}=1$
$\alpha_{os\rightarrow sarc}=1$	$\alpha_{sarc\rightarrow bone}=1$

Video S2. Simulation of osteosarcoma structure propagation from a bone inoculation (with above parameters).
